# Hospital Sites

**Published:** 1920-07-03

**Authors:** 


**THE PUBLIC WELL-BEING**—(*continued*)

### Hospital Sites.

### Points About the Removal Suggestion.

The renewal of the proposal that consideration should be given to the possibility of removing the London hospitals from their present sites to the country has not met with much response, though it comes at a moment when the whole position of the hospitals is the subject of growingly acute controversy. About seventeen years ago it was debated by the Hospitals' Association, which considered the problem from two standpoints—ought any of the large hospitals to be moved (1) to sites in more congested districts, or (2) to sites in the country. Sir Trevor Lawrence was in the chair, and the

THE PUBLIC WELL-BEING—(*continued*).

discussion was based on a paper by Sir Henry Burdett. Sir Henry reviewed the needs of some of the more congested areas, and had one or two proposals to make for removal to meet the needs. But the removal from cities to the country he considered to be impracticable, if due regard were had to the interests of the patients and the circumstances which affected city populations. His view was similar to that recently expressed by the secretary of the London Hospital—namely, that the extension of convalescent hospitals situated in the country represents the best form in which large hospitals can keep their patients in touch with the country without incurring grave risks of adding to the sufferings and anxieties of the sick and their families in large centres of population.

The discussion which followed was generally in accord with this view. It was pointed out that it was necessary to remember hospitals were voluntarily supported, and removals might have serious effects on subscriptions made for particular needs. Another consideration put forward was that it was necessary to economise the time of the distinguished physicians and surgeons who gave their services to the great hospitals. It might be found, on removal, that it was scarcely possible for eminent men to continue those services. It was also argued that it would actually entail a great sacrifice of money which could not be faced, even in the mere expenditure of removal. This is interesting, because the general idea of the latest proposal is that the sale of the valuable sites would be profitable.

The Secretary of the Royal Free Hospital entered a plea for a trial. He considered there was a growing belief that a large proportion of patients would do much better if they were treated in the pure air of the country instead of in the dense atmosphere of London. Medical men knew very well that it often happened that a week or two after an operation a patient began to flag, and that in those circumstances the best remedy was to send him away into the country. That was generally done by sending such patients to a convalescent home in the country, whence they were frequently sent back to London to undergo still further treatment. This was a very unsatisfactory state of things. Instead of drafting patients to London they ought to be able to send them to the country, and make provision for their proper and effective treatment while there. In Paris they were already finding the need for doing something in that connection. (This refers to 1903.) The great Hôtel Dieu and other of the City hospitals were to be removed, and large hospitals built, at a cost of something like three and a half millions, in the suburbs. In Berlin action was being taken on the same lines, and he did not think they in London should allow themselves to be behind in the way of building at least one large hospital in the country, in order that their medical men might judge for themselves by comparison as to the relative benefit derived by the patients in a pure atmosphere.

The consensus of opinion seemed, however, to be in favour of the continuation of the arrangement whereby the city hospitals remained on the existing sites, accessible to the big population, a centre for difficult cases from all parts of the land, and with convalescent homes for recuperation in the country. Apart from the consideration of the proposal as a matter of policy, the financial element is a big one, and would require very careful and exhaustive inquiry before it became possible to express a final opinion.

